# Psychological Profile, Competitive Anxiety, Moods and Self-Efficacy in Beach Handball Players

**DOI:** 10.3390/ijerph17010241

**Published:** 2019-12-29

**Authors:** Rafael E. Reigal, Juan A. Vázquez-Diz, Juan P. Morillo-Baro, Antonio Hernández-Mendo, Verónica Morales-Sánchez

**Affiliations:** Faculty of Psychology, University of Malaga, Teatinos Campus, 29071 Malaga, Spain; rafareigal@uma.es (R.E.R.); javazquezdiz@gmail.com (J.A.V.-D.); juanpablo.morillo@gmail.com (J.P.M.-B.); vomorales@uma.es (V.M.-S.)

**Keywords:** psychological skills, sports performance, team sport

## Abstract

The first objective of this research was to analyze the relationships between the sports psychological profile, competitive anxiety, mood and self-efficacy in beach handball players. The second objective was to determine the predictive capacity of the psychological profile on competitive anxiety, moods and self-efficacy, which was assessed by linear regression analysis. One hundred and eighty-one beach handball players participated in this research (age: *M* = 25.68; *SD* = 5.95), of which 52.49% were male (*n* = 95) and 47.51% were female (*n* = 86). The Psychological Sports Execution Inventory (SPPI), the Competition Anxiety State Inventory 2 (CSAI-2), the Mood Profile (POMS) questionnaire and the General Self-Efficacy Scale (GSES) were used to obtain the data. Correlation and linear regression analyses reveal statistically significant associations between the constructs studied, both for the total sample and by gender. Specifically, they highlight the relationships between the different measures of the sports psychological profile with self-confidence (*p* < 0.001), as well as those established between negative coping control with competitive anxiety (*p* < 0.001), moods (*p* < 0.05) and general self-efficacy (*p* < 0.001).

## 1. Introduction

The analysis of psychological factors that may influence the performance of athletes has been a subject of study in recent decades in numerous disciplines [1,2,3]. There is a growing understanding of how aspects such as competitive anxiety, motivation, moods, flow states, and self-efficacy relate to each other [4,5]. Thus, in the training processes of athletes, the assessment of their psychological functioning is a dimension that is increasingly being integrated into their global preparation [6,7].

Among the most relevant psychological manifestations linked to sports performance is competitive state anxiety, which must be controlled to improve the processes of adaptation to competition and is currently the object of interest of multiple researchers [8,9,10]. The factors that can influence competitive state anxiety, both in its cognitive and somatic manifestation and according to the model proposed by Martens and collaborators [11,12,13], are diverse, such as the perception of opponent level, the importance of the competition, the confidence in the own capacities or the abilities of coping with the stress [14,15].

Another of the psychological constructs that have been analyzed in depth in the sports field are moods, highlighting how some dimensions could influence the performance of athletes [16,17]. One of the most commonly used questionnaires to analyze moods is the one proposed by McNair, Lorr and Dorppleman [18], called the Profile of Mood States (POMS), which considers seven factors: tension, depression, anger, vigor, fatigue, confusion and friendship. In a sporting context, it has been observed in numerous works that a specific score of its dimensions (known as the iceberg profile) is a good predictor of sports performance [19,20,21]. This profile implies higher scores in the vigor dimension and lower scores in the other elements, as has been shown in previous literature [21,22].

Likewise, self-efficacy refers to the judgments that people make about their ability to perform a task [23,24]. Self-efficacy is considered a behavior modulator and develops in people from previous successes in past behaviors, vicarious experiences, verbal persuasion or the physiological states presented [23,25]. Some authors have emphasized the need to assess specific measures of self-efficacy [23], but others point out that a general perception of self-efficacy may be an adequate predictor of behavior [26]. Self-efficacy has been deeply analyzed in the sports field, as one of the psychological constructs considered most relevant to athletic performance [27,28,29].

On the other hand, it is considered that the athlete can manage a series of psychological skills which are necessary to better adapt to competition contexts. To evaluate these capacities, there are instruments such as the Psychological Inventory of Sports Performance (SPPI) [30,31], which allows the analysis of a wide range of factors such as self-confidence, negative coping control, attentional control, visuo-imaginative control, motivational level, positive coping control and attitudinal control. This instrument has been widely used for the Spanish-speaking population in recent years, and has been sensitive to establishing differences between athletes and their relationship with other psychological constructs linked to sports performance [32,33,34].

Several studies have analyzed the relationships between the variables included in the sports psychological profile [30,35,36,37,38,39,40] with competitive anxiety, moods and self-efficacy. In the sporting context, assessing the association between these variables is relevant given their link to competitive performance. Thus, some investigations have addressed this phenomenon in various sports. Reigal, Delgado Giralt, López-Cazorla, and Hernández-Mendo [35] analyzed a group of adult triathletes, observing negative relationships between psychological skills such as coping control or attentional control with cognitive and somatic anxiety, as well as positive relationships between these skills and self-confidence. Verner-Filion et al. [36] observed in a group of adolescent athletes that inadequate coping strategies were related to greater cognitive anxiety. Likewise, Covassin and Pero [37] observed in young tennis players that those who showed greater self-confidence and lower levels of anxiety were able to better face negative events.

Lane, Jones, and Stevens [38] analyzed a group of young tennis players and observed that adaptive coping strategies are essential to protect perceptions of self-efficacy against negative events in competition. On the other hand, Besharat, and Pourbohlool [39] analyzed 246 athletes and observed that self-confidence was negatively related to somatic and cognitive anxiety and positively related with perceptions of sports self-efficacy. Peñaloza, Jaenes, Méndez-Sánchez, and Jaenes-Amarillo [40] evaluated 255 adult athletes from different disciplines and observed that self-confidence positively predicted the vigor and negatively the confusion of the Profile of Mood States questionnaire.

Sports psychology has studied different psychological constructs in handball [2,41,42,43,44]. However, the beach handball modality has been little explored because it is a relatively recent discipline. The few investigations carried out have analyzed some psychological variables, pointing out the relationships between the perception of motivational orientation and autonomous support with basic psychological needs [45,46] or the association between the sports psychological profile with competitive anxiety and self-confidence [47]. Even so, the data obtained could not be contrasted with other researches that could consolidate the findings obtained. 

Thus, research in this sport should continue to increase its evidence to better understand the behavior of athletes and establish strategies for their training. In addition, although handball and beach handball present similarities, there are features that make them different sports [48]. As an example, in beach handball, goals of single and double value (specialist throws, flight throws or 360-degree spin throws) can be obtained [49]. In this way, the game is different in both modalities, and requires the analysis of the athlete in a specific way.

Therefore, given the need to obtain data to better understand beach handball players and how they relate to various psychological constructs related to sports performance, the aim of this study was to determine the relationships between the psychological skills proposed by the Psychological Sports Profile Inventory (SPPI) with competitive anxiety, moods and self-efficacy in a sample of beach handball players. Likewise, this study aimed to determine the predictive capacity of psychological profile on competitive anxiety, moods and self-efficacy was assessed by linear regression analysis.

## 2. Materials and Methods 

### 2.1. Design

A comparative and predictive design was used to carry out this research [50]. It is a cross-sectional design, in which correlation measures and predictive models were applied to contrast the research objectives. For this, a single evaluation was carried out for data collection, which were subsequently statistically processed.

### 2.2. Participants

The study included 181 (52.49% male, 47.51% female) senior beach handball players (age: *M* = 25.68; *SD* = 5.95) during the 2016 Spanish Cup, a high-level national competition in which only teams classified according to the previous year’s ranking participate.

### 2.3. Materials and Measures

#### 2.3.1. Sport Performance Psychological Inventory (SPPI)

This questionnaire was developed by Hernández-Mendo [30]. It is based on Loehr’s Psychological Performance Inventory (PPI) [51,52] and was built to assess the psychological abilities of athletes. This instrument has shown an adequate composite reliability (>0.71), average extracted variance (≥0.57), and convergent and discriminant validity; furthermore, its factorial structure has been confirmed [31]. It consists of 42 items and seven factors: self-confidence, negative coping control, attentional control, visuo-imaginative control, motivational level, positive coping control and attitudinal control. The results were evaluated using a Likert scale from one (almost never) to five (almost always). The internal consistency values (Cronbach’s Alpha) for this research were as follows: self-confidence = 0.72, negative coping control = 0.71, attentional control = 0.65, visuo-imaginative control = 0.76, motivational level = 0.64, positive coping control = 0.71 and attitudinal control = 0.69.

#### 2.3.2. Competitive State Anxiety Inventory-2 (CSAI-2) 

This questionnaire was proposed by Martens et al. [13] and assesses competitive anxiety and self-confidence, using 27 items and three factors: cognitive anxiety, somatic anxiety and self-confidence. In this investigation, the Spanish version developed by Capdevila [53] was used. In the Spanish sample, its validity and reliability have been contrasted, and its three-factor factor structure has been confirmed and with internal consistency values for each dimension greater than 0.71 [54]. The results were evaluated using a Likert scale from one (almost never) to five (almost always). The internal consistency values (Cronbach’s Alpha) for this research were as follows: cognitive anxiety = 0.61, somatic anxiety = 0.82 and self-confidence = 0.83.

#### 2.3.3. Profile of Mood States Questionnaire (POMS) 

This questionnaire was proposed by McNair, Lorr and Droppleman [18]. In this investigation, the Spanish version developed by Arce, Andrade and Seoane [55] was used. In that study, the factorial structure was analyzed, obtaining seven factors, and adequate internal consistency values were obtained (≥0.70). It is a list composed of 65 adjectives, initially designed for the clinical field and later extended to other areas. This instrument evaluates the dimensions of tension, depression, anger, vigour, fatigue, confusion and friendship. The results were evaluated using a Likert scale between 0 (nothing) and four (very much). The internal consistency values (Cronbach’s Alpha) for this research were as follows: depression = 0.85, vigor = 0.70, anger = 0.87, stress = 0.75 and fatigue = 0.86.

#### 2.3.4. General Self-Efficacy Scale (GSES) 

This questionnaire was proposed by Schwarzer and Jerusalem [56]. In this investigation, the Spanish version developed by Baessler and Schwarzer [57] was used. Its validity and reliability have been contrasted in a Spanish sample, showing an adequate internal consistency value (0.87). This scale, made up of 10 items, measures the stable feeling of competence to handle a wide range of life situations. The result was evaluated using a Likert type response scale between 0 (strongly disagree) and 10 (strongly agree). The internal consistency value (Cronbach’s Alpha) for this research was 0.92.

### 3.4. Procedure

In order to obtain the participation of the sportsmen and women, the research team met with the heads of the sports clubs, requesting the participation of their players. After agreement with the teams, informed consent was obtained from the players and it was explained to them how and when the data would be taken. The information was collected 24 h before the start of the competition in a room set up for this purpose (first match played in the 2016 Spanish Cup). The questionnaires were completed only once, after which the data were codified and statistically processed. Participants filled out the questionnaires individually, although possible doubts were resolved. It was indicated that there were no good or bad answers, but the important thing was that they should respond as they thought. It took approximately 45 to 60 min to answer all the questionnaires, and all were completed in one session. As the questionnaires were filled out, the participants raised their hands and the papers were collected. After that, everyone could leave the room. The instruments used were in Spanish, so questionnaires were used whose validity and reliability had been analyzed favorably and previously in this language [30,31,53,54,55,56]. The ethical principles of the Declaration of Helsinki [58] were respected throughout the research process and the research was approved by the Ethics Committee of the University of Malaga (no. 243, CEUMA Registry No. 18-2015-H).

### 3.5. Data Analysis

The data were subjected to descriptive and inferential analysis. The normality of the data was checked through skewness, kurtosis and the Kolmogorov–Smirnov test. Normal distribution was considered if the skewness showed values between −2 and 2, and kurtosis between −7 and 7; besides that, the Kolmogorov–Smirnov test is not significant [59]. In order to adjust the sample to normal, the data of each variable were adjusted using ln(x) and x^2^ if they were not normally distributed. The internal consistency of the different scales was estimated using Cronbach Alpha coefficient. Pearson’s bivariate coefficient was used to analyze the correlations between the factors (±0.01 to ±0.19 = very weak correlation; ±0.20 to ±0.39 = weak correlation; ±0.40 to ±0.59 = moderate correlation; ±0.60 to ±0.79 = high correlation [60]). The predictive capacity of psychological profile on competitive anxiety, moods and self-efficacy was assessed by linear regression analysis (successive steps). The IBM SPSS Statistics v20 software package (IBM Corp, Armonk, NY, USA) has been used for the statistical processing of the data.

## 3. Results

### 3.1. Descriptive Analysis and Data Normality

Table 1 and Table 2 show the descriptive statistics of the variables under study, as well as the results of the normality tests (Kolmogorov–Smirnov). As can be observed, the dimensions of motivational level, positive coping control, vigor, depression, anger and fatigue showed problems of normality. These variables were adjusted with equivalent measurements generated by the ln(x) and x^2^ algorithms.

### 3.2. Correlations and Linear Regression

Table 3 shows the correlations (Pearson) established between the dimensions of the SPPI with those of the CSAI-2, POMS and GSES. In general, the data showed statistically significant associations between most factors, considering the total sample and according to gender. The relationships established with self-confidence (CSAI-2), vigor (POMS) and general self-efficacy (GSES) are positive, with the rest of the cases resulting as negative, except for the relationship between motivational level and cognitive anxiety (in the total sample and in the female category), which is also positive. For both the general sample and according to gender, self-confidence (CSAI-2) is the variable that shows the highest level of correlation with SPPI variables. By gender, the male category has slightly higher correlations than the female category. For the total sample and for the male category, the visuo-imaginative control and the motivational level (SPPI) are the factors that show a lower level of correlation with the rest of the variables. For the female category, it is the visuo-imaginative control (SPPI) that shows the lowest level of correlation with the dimensions of the CSAI-2 and POMS. 

Table 4 and Table 5 show the linear regression analyses performed (successive steps), with the predictor variables in each model being the dimensions of the *P* and the criterion variables being each factor of CSAI-2, POMS and GSES. The most complete model offered by the analyses is shown. The variables excluded in the various cases are not present due to a lack of significance (*p* > 0.05). The SPPI self-confidence variable has been excluded for the analysis of CSAI-2 self-confidence and GSES self-efficacy due to the similarity of constructs [30]. The data meet the linearity assumptions in the relationship between predictor variables and criteria, as well as the homoscedasticity and normal distribution of waste, whose mean value is 0 and the standard deviation is practically 1 (0.99). In addition, Durbin–Watson values are satisfactory since they range between 1.56 and 2.34 [61]. 

For the total sample (Table 4), the results indicate that an explanatory model has been generated for each variable of the CSAI, POMS and GSES which explains between 17% and 22% of the variance, except for somatic anxiety and self-confidence (CSAI-2). The model for somatic anxiety explains 31% of the variance and includes the predictor negative coping control (*R* = 0.56; *R*^2^ adjusted = 0.31; *F* = 80.21; *p* < 0.001). For self-confidence (CSAI-2), the model explains 44% of the variance and includes the predictors positive coping control, attentional control and attitudinal control (*R* = 0.67; *R*^2^ adjusted = 0.44; *F* = 47.40; *p* < 0.001). 

By gender (Table 5), the results indicate that an explanatory model has been generated for each CSAI, POMS and GSES variable that explains between 12% and 28% of the variance, except for somatic anxiety in the male category and self-confidence for both categories (CSAI-2). The model for somatic anxiety in the male category explains 36% of the variance and includes the predictor negative coping control (*R* = 0.61; *R*^2^ adjusted = 0.36; *F* = 52.86; *p* < 0.001). For self-confidence (CSAI-2) the model explains 41% of the variance for both categories. The male category includes attentional control and positive coping control predictors (*R* = 0.65; *R*^2^ adjusted = 0.41; *F* = 32.99; *p* < 0.001). For the female category, the model includes the attitudinal control and attentional control variables (*R* = 0.65; *R*^2^ adjusted = 0.41; *F* = 30.14; *p* < 0.001).

## 4. Discussion

The objective of this work was twofold. On the one hand, the aim of the study was to determine the relationships between the psychological profile of a sample of beach handball players with levels of competitive anxiety, moods and general self-efficacy. On the other hand, this research tried to determine the predictive capacity of the psychological profile on the other psychological variables. The results have shown statistical significant associations among the studied constructs and a remarkable predictive capacity of the psychological abilities of athletes on competitive anxiety, moods and general self-efficacy. Furthermore, although there are slight differences between men and women, the results offer quite a few similarities in both genders.

The data provided in this research have few precedents in the field of beach handball. Partially, some relationships proposed in this work had been studied, such as those found in the study of Morillo, Reigal and Hernández-Mendo [47]. However, the relationships between the set of constructs proposed in this research had not been analyzed, and they were also differentiated by gender. For this reason, the obtained results make a significant contribution to beach handball and allow us to better understand psychological aspects that have been considered relevant for athletes’ performance in previous investigations [8,9,16,29].

Firstly, correlation analyzes show negative relationships between some psychological abilities, such as self-confidence, negative coping control or attentional control, and competitive anxiety in the total sample and by gender. This is consistent with previous work [62,63]. Likewise, we can see how sports psychological skills are clearly positively related to self-confidence in the athletes analyzed, coinciding with previous works [35,47]. In almost all cases, they are moderate, but some approach high correlations. Specifically, in beach handball, Morillo et al. [47] observed similar results to those obtained in this study, although by categories, women obtained a lower relationship between negative coping control and cognitive and somatic anxiety. Likewise, in the work of Morillo et al. [47], attentional control was also significant, but it was only related to somatic anxiety. In any case, the main aspect that these results suggest is that training and improving aspects such as greater attention control or developing strategies to better assimilate events that are perceived as negative could help athletes to face the competition with more confidence and a better feeling of competitive anxiety [64,65].

Statistical significant associations are also seen between sports psychological abilities and different measures of mood in athletes. In general, it is observed that psychological skills are negatively associated with negative dimensions of mood and positively associated with vigor. Specifically, athletes’ moods help identify whether athletes are adapting to training loads and competition. Therefore, mood indicators could detect processes such as over-training or problems to adapt psychologically to sports stressors [66]. Likewise, these data suggest that a good development of aspects included in the Sport Performance Psychological Inventory could contribute to a lower development of negative feelings and an increase in adaptive moods. Thus, coaches could use these indicators to assess how an athlete is approaching competition and help them.

Regarding general self-efficacy, the data have highlighted that it is positively associated with the sports psychological profile. This is very relevant for sports performance, because this construct is considered a psychological variable that is linked to sports performance. Likewise, although specific self-efficacy measures have been used in many previous investigations [67,68], several authors consider that general self-efficacy, which would refer to the perception of being effective in a broad set of situations, would be a suitable factor for predicting behavior [69]. As the results found indicate, general self-efficacy has been associated with different factors of the Sport Performance Psychological Inventory. Therefore, we believe that the development of psychological skills could have an impact on the improvement of the perception of an athlete to handle various situations, among which would be those directly related to competition and training.

Secondly, linear regression analyses show that the sports psychological profile adequately predicts anxiety and self-confidence scores. Among these factors, it is observed that coping control (positive and negative) as well as attentional and attitudinal control are important predictors in these models. By gender, regression models for cognitive anxiety present differences, with the attentional control appearing in the male category as the best predictor, and for the female category, the negative coping control is accompanied by the motivational level. This would indicate the importance of training the psychological skills of the athlete, especially coping strategies for negative situations, as one of the tools the athlete could develop to avoid the development of competitive anxiety states [64,65]. If these psychological variables can predict scores in anxiety and self-confidence, it would be interesting for coaches to make an effort to train them, given their influence on the performance of athletes.

We must highlight the similarity of this work with that of Morillo et al. [47], given that negative coping control and motivational level are predictors of anxiety levels. Specifically, they agree to our pointing out the motivational level in womenas a predictor of cognitive anxiety. These results may indicate that women are less able to regulate their cognitive activation before a competition, which affects the levels of cognitive anxiety presented. This difference shown by the data could have repercussions on the way athletes are prepared, taking this data into account in the female category in order to try to modulate their previous level of motivation and help reduce the cognitive anxiety presented. Previous work has analyzed this phenomenon and has highlighted that intensity in motivational levels could produce an increase in anxiety [70].

Morillo et al. [47] observed that coping control in men as well as attitudinal control and attentional control in women were the best predictors of self-confidence. Although, in the present study, the predictors in meninclude positive coping and attitudinal control, in general, it is observed that the variables that have best been related to self-confidence in the group of participants are coping control, attitudinal control and attentional control. These psychological skills refer to a series of abilities to establish action strategies, maintain attention and concentration or regulate behavior [30], so it is congruent that those athletes who perceive a high skill in these skills feel more prepared to face their sporting task. Specifically, attitudinal control has been positively related to athletic performance and is considered a skill present in athletes with higher levels of performance [32,71]. In this sport, as in tennis or volleyball, the set scoring system magnifies the importance of attitude control as a psychological skill [47].

In addition, linear regression models have highlighted that some Sport Performance Psychological Inventory factors have been predictors of mood scores. In both categories, aspects such as self-confidence or coping control, both positive and negative, appear as significant variables in the prediction of mood profile negative factor scores. In girls, for the prediction of anger and fatigue, attitudinal control emerges as a significant variable. The results found could indicate that the development of psychological skills in sportsmen and women would help them to have better emotional sensations linked to competition, and to prepare them to better face the challenges they face [72]. Thus, this is something that coaches should take into account given the relationship that has been observed between moods and athletic performance [66].

Besides this, although there are slight differences in the predictive capacity of each dimension of the psychological profile regarding the perception of general self-efficacy, one can appreciate how there is a notable relationship between the constructs. In this sense, the perception of general self-efficacy could be a good measure to assess the possible behavior of athletes [73,74]. Beyond sporting expertise, players during competition must face different processes of adaptation to the changing environment in which this sport is developed, weather conditions and stays away from home, and must be resilient to failure or must perform their task in contexts under pressure. Therefore, specific physical self-efficacy measures are appropriate to assess the perception of sports skills, but general self-efficacy may provide relevant information to predict the athlete’s behavior in a broad competitive context.

In conclusion, the results found in this work indicate the importance of including the training of psychological skills in the training processes of athletes. As the data presented suggest, the development of certain skills may allow the improvement of some psychological parameters that have been related to sports performance. Specifically, the mental preparation of the athlete must be a fundamental part in the training processes of beach handball players, given that it is a sport in which it is necessary to face situations of great uncertainty such as those endured at the beginning of the second set or the final tiebreaker situation (shoot-out throws). 

This work presents a series of limitations, highlighting the scarcity of studies of this nature carried out in beach handball. For that reason, it is necessary to continue exploring this modality, and finding the similarities or differences that can present with the modality of handball indoor. In addition, it would be necessary to carry out this type of research in different categories and levels of competition, to observe if the obtained results are stable and specific patterns can be established. To delve deeper into these aspects would allow us to offer more contrasted indications to the technicians of this sport so that they can work with greater guarantees of success in the preparation of their players. However, we consider this study valuable because it provides non-existent data in this sport and helps to better understand the relationships between various psychological variables that could affect the beach handball player.

## 5. Conclusions

This study highlights the relationships among measures of the sports psychological profile with self-confidence, as well as between coping control and competitive anxiety, moods and general self-efficacy. Thus, the sports psychological profile is able to predict competitive anxiety, moods and self-efficacy scores, and coping control is one of the most relevant dimensions in these predictive models. Likewise, self-confidence is the variable that best predicts the sports psychological profile, specifically through positive coping control, attitudinal control and attentional control.

## Figures and Tables

**Table 1:** Means and typical deviations of Psychological Inventory of Sports Performance (SPPI), Competitive State Anxiety Inventory-2 (CSAI-2), Profile of Mood States Questionnaire (POMS) and General Self-Efficacy Scale (GSES) scores.

Study Variables	Total		Male (n = 95)		Female (n = 86)	
	*M*	*SD*	*M*	*SD*	*M*	*SD*
SPPI						
Self-confidence	3.90	0.65	4.04	0.65	3.74	0.61
Coping control (−)	3.37	0.65	3.42	0.61	3.32	0.68
Attentional control	3.44	0.52	3.52	0.52	3.36	0.51
Visuo-imaginative control	3.66	0.71	3.75	0.63	3.56	0.77
Motivational level	4.01	0.58	4.08	0.57	3.93	0.58
Coping control (+)	4.02	0.58	4.12	0.59	3.92	0.55
Attitudinal control	3.95	0.55	4.01	0.54	3.89	0.55
CSAI-2						
Cognitive anxiety	3.48	0.42	3.46	0.43	3.49	0.42
Somatic anxiety	2.58	0.72	2.60	0.68	2.55	0.77
Self-confidence	3.87	0.56	4.01	0.55	3.71	0.54
POMS						
Tension	1.72	0.84	1.73	0.87	1.70	0.81
Vigor	2.91	0.68	2.92	0.66	2.89	0.70
Depression	0.62	0.81	0.67	0.82	0.56	0.79
Cholera	1.14	0.77	1.19	0.78	1.08	0.77
Fatigue	1.16	0.93	1.13	0.92	1.20	0.95
GSES						
General self-efficacy	7.82	1.36	8.09	1.28	7.53	1.40

**Table 2:** Normality analysis of SPPI, CSAI-2, POMS and GSES scores.

Study Variables	Total			Male (95)			Female (86)		
	*S*	*K*	*K-S*	*S*	*K*	*K-S*	*S*	*K*	*K-S*
SPPI									
Self-confidence	−0.39	−0.20	1.29	−0.71	0.52	1.12	−0.14	−0.46	0.89
Coping control (-)	0.04	−0.50	1.08	0.13	−0.73	1.07	0.02	−0.40	0.90
Attentional control	−0.15	0.27	1.20	−0.37	0.95	0.96	0.08	−0.10	1.04
Visuo-imaginative control	−0.24	−0.40	1.10	−0.24	−0.13	0.80	−0.12	−0.68	0.85
Motivational level	−0.52	−0.29	1.55 *	−0.79	0.02	1.50 *	−0.25	−0.28	0.76
Coping control (+)	−0.36	−0.71	1.52 *	−0.52	−0.68	1.45 *	−0.26	−0.56	0.97
Attitudinal control	−0.19	−0.33	1.05	−0.32	0.13	0.89	−0.06	−0.66	0.84
CSAI-2									
Cognitive anxiety	−0.04	0.06	1.04	0.08	−0.65	0.98	−0.17	1.03	0.79
Somatic anxiety	0.14	−0.46	0.73	0.11	−0.61	0.68	0.19	−0.39	0.60
Self-confidence	−0.37	0.10	1.08	−0.51	0.27	0.83	−0.35	0.27	0.98
POMS									
Tension	0.15	−0.86	1.28	0.17	−0.85	0.88	0.11	−0.88	0.99
Vigor	−0.94	1.14	1.71 **	−0.94	1.34	1.12	−0.94	1.06	1.31
Depression	1.37	0.95	2.99 ***	1.27	0.61	2.04 ***	1.53	1.56	2.41 ***
Cholera	0.89	−0.19	2.11 ***	0.72	−0.50	1.31	1.11	0.35	1.73 **
Fatigue	0.73	−0.03	1.43 *	0.79	0.10	1.39 *	0.68	−0.09	1.09
GSES									
General self-efficacy	0.42	5.53	1.26	1.68	11.94	1.11	−0.51	−0.02	0.93

Note: S = Skewness; K = Kurtosis; K-S = Kolmogorov–Smirnov. * *p* < 0.05; ** *p* < 0.01; *** *p* < 0.001.

**Table 3:** Analysis of correlations between SPPI with CSAI-2, POMS and GSES.

Study Variables	CSAI-2			POMS					GSES
	COG	SOM	SELF-C	T	V	D	C	F	GSE
SPPI (Total)									
Self-confidence	−0.13	−0.19 *	0.59 ***	−0.22 **	0.27 ***	−0.36 ***	−0.30 **	−0.31 ***	0.37 ***
Coping control (-)	−0.30 ***	−0.53 ***	0.50 ***	−0.39 ***	0.15 *	−0.24 **	−0.37 ***	−0.28 ***	0.26 ***
Attentional control	−0.28 ***	−0.29 ***	0.53 ***	−0.27 ***	0.10	−0.25 **	−0.30 ***	−0.27 ***	0.24 **
Visuo-imaginative control	0.02	0.06	0.31 ***	0.06	0.21 **	−0.14	−0.06	−0.08	0.34 ***
Motivational level	0.16 *	0.01	0.39 ***	−0.01	0.32 ***	−0.21 **	−0.12	−0.15 *	0.33 ***
Coping control (+)	0.05	−0.13	0.60 ***	−0.20 **	0.28 ***	−0.34 ***	−0.34 ***	−0.31 ***	0.38 ***
Attitudinal control	−0.02	−0.22 **	0.57 ***	−0.27 ***	0.29 ***	−0.24 **	−0.29 ***	−0.23 **	0.42 ***
SPPI (Male)									
Self-confidence	−0.20 *	−0.22 *	0.54 ***	−0.24 *	0.28 **	−0.44 ***	−0.44 ***	−0.27 **	0.36 ***
Coping control (-)	−0.32 **	−0.57 ***	0.46 ***	−0.39 ***	0.18	−0.35 ***	−0.41 ***	−0.26 *	0.31 **
Attentional control	−0.41 ***	−0.31 **	0.49 ***	−0.32 **	0.16	−0.31 **	−0.39 ***	−0.23 *	0.18
Visuo-imaginative control	−0.02	−0.07	0.33 ***	−0.04	0.37 ***	−0.27 **	−0.16	−0.04	0.32 **
Motivational level	0.01	−0.05	0.36 ***	0.02	0.31 **	−0.24 *	−0.21 *	−0.02	0.37 ***
Coping control (+)	0.02	−0.16	0.62 ***	−0.22 *	0.25 *	−0.38 ***	−0.43 ***	−0.24 *	0.33 **
Attitudinal control	−0.05	−0.29 **	0.55 ***	−0.29 **	0.29 **	−0.28 **	−0.28 **	−0.12	0.34 ***
SPPI (Female)									
Self-confidence	−0.03	−0.18	0.60 ***	−0.23 *	0.18	−0.33 **	−0.38 ***	−0.28 **	0.33 **
Coping control (-)	−0.27 *	−0.51 ***	0.53 ***	−0.40 ***	0.09	−0.15	−0.44 ***	−0.27 *	0.19
Attentional control	−0.14	−0.29 **	0.53 ***	−0.23 *	0.06	0.01	−0.34 **	−0.23 *	0.25 *
Visuo-imaginative control	0.05	0.16	0.24 *	0.14	0.08	−0.15	0.01	0.05	0.32 **
Motivational level	0.35 ***	0.02	0.39 ***	−0.09	0.35 **	−0.26 *	−0.18	−0.30 **	0.23 *
Coping control (+)	0.11	−0.13	0.54 ***	−0.17	0.31 **	−0.34 **	−0.39 ***	−0.33 **	0.40 ***
Attitudinal control	0.02	−0.16	0.58 ***	−0.24 *	0.22 *	−0.20	−0.40 ***	−0.38 ***	0.47 ***

Note: SPPI = Sport Performance Psychological Inventory; CSAI-2 = Competitive State Anxiety Inventory-2; POMS = Profile of Mood States; GSES = General Self-Efficacy Scale; COG = Cognitive Anxiety; SOM = Somatic Anxiety; AUT = Self-Confidence; T = Tension; V = Vigor; D = Depression; C = Anger; F = Fatigue; GSE = General Self-Efficacy. * *p* < 0.05; ** *p* < 0.01; *** *p* < 0.001.

**Table 4:** Linear regression analysis (successive steps) for the total sample.

Criterion	*R*	*R^2^*	*D-W*	Predictors	*Beta*	*t*	*T*	*VIF*
Cognitive anxiety	0.43	0.17	2.26	Coping control (-)	−0.238	−2.77 ***	0.62	1.61
				Motivational level	0.300	4.20 ***	0.90	1.11
				Attentional control	−0.227	−2.62 ***	0.61	1.64
Somatic anxiety	0.56	0.31	1.85	Coping control (-)	−0.563	−8.96 ***	1.00	1.00
Self-confidence	0.67	0.44	2.03	Coping control (+)	0.287	3.48 ***	0.46	2.17
				Attentional control	0.258	3.85 ***	0.70	1.44
				Attitudinal control	0.244	3.04 **	0.49	2.05
Tension	0.46	0.21	1.79	Coping control (-)	−0.462	−6.82 ***	1.00	1.00
Vigor	0.48	0.22	1.85	Motivational level	0.356	4.54 ***	0.75	1.34
				Attitudinal control	0.184	2.35 *	0.75	1.34
Depression	0.46	0.20	1.75	Self-confidence	−0.275	−2.81 **	0.48	2.09
				Coping control (+)	−0.222	−2.26 *	0.48	2.09
Cholera	0.46	0.20	2.12	Coping control (-)	−0.323	−0.404 ***	0.72	1.39
				Self-confidence	−0.196	−2.46 *	0.72	1.39
Fatigue	0.43	0.17	2.18	Self-confidence	−0.317	−3.93 ***	0.74	1.34
				Coping control (-)	−0.167	−2.07 *	0.74	1.34
General self-efficacy	0.46	0.20	2.14	Attitudinal control	0.248	2.83 **	0.60	1.67
				Motivational level	0.168	2.18 *	0.77	1.29
				Coping control (-)	0.162	2.03 *	0.72	1.39

Note: M = Model; D-W = Durbin–Watson; T = Tolerance Index; VIF = Variance Inflation Factor. * *p* < 0.05; ** *p* < 0.01; *** *p* < 0.001.

**Table 5:** Linear regression analysis (successive steps) by gender.

Criterion.	Category	*R*	*R* ^2^	*D-W*	Predictors	*Beta*	*T*	*T*	*VIF*
Cognitive anxiety	Male	0.51	0.25	2.25	Attentional control	−0.510	−5.59 ***	1.00	1.00
	Female	0.49	0.22	2.16	Motivational level	0.413	4.25 ***	0.97	1.03
					Coping control (-)	−0.345	−3.55 ***	0.97	1.03
Somatic anxiety	Male	0.61	0.36	2.25	Coping control (-)	−0.608	−7.27 ***	1.00	1.00
	Female	0.52	0.27	1.56	Coping control (-)	−0.518	−5.52 ***	1.00	1.00
Self-confidence	Male	0.65	0.41	2.10	Attentional control	0.233	2.49 *	0.73	1.38
					Coping control (+)	0.493	5.28 ***	0.73	1.38
	Female	0.65	0.41	1.96	Attitudinal control	0.427	4.42 ***	0.75	1.34
					Attentional control	0.318	3.29 **	0.75	1.34
Tension	Male	0.46	0.20	1.88	Coping control (-)	−0.455	−4.82 ***	1.00	1.00
	Female	0.47	0.21	1.64	Coping control (-)	−0.473	−4.81 ***	1.00	1.00
Vigor	Male	0.48	0.21	1.66	Attitudinal control	0.322	3.17 **	0.85	1.17
					Visuo-imaginative control	0.252	2.49 *	0.85	1.17
	Female	0.46	0.20	2.25	Motivational level	0.355	3.66 ***	1.00	1.00
Depression	Male	0.50	0.24	1.66	Self-confidence	−0.500	−5.44 ***	1.00	1.00
	Female	0.38	0.14	1.86	Coping control (+)	−0.384	−3.79 **	1.00	1.00
Cholera	Male	0.54	0.28	2.10	Coping control (-)	−0.295	−2.84 **	0.74	1.35
					Coping control (+)	−0.318	−3.15 **	0.74	1.35
	Female	0.49	0.22	2.15	Coping control (-)	−0.316	−2.86 **	0.75	1.33
					Attitudinal control	−0.245	−2.22 *	0.75	1.33
Fatigue	Male	0.36	0.12	2.34	Self-confidence	−0.363	−3.68 ***	1.00	1.00
	Female	0.41	0.16	1.93	Attitudinal control	−0.409	−3.98 ***	1.00	1.00
General self-efficacy	Male	0.50	0.24	2.24	Coping control (-)	0.358	3.70 ***	0.88	1.14
					Motivational level	0.250	2.58 *	0.88	1.14
	Female	0.49	0.23	1.99	Attitudinal control	0.493	5.17 ***	1.00	1.00

Note: M = Model; D-W = Durbin–Watson; T = Tolerance Index; VIF = Variance Inflation Factor. * *p* < 0.05; ** *p* < 0.01; *** *p* < 0.001.
